# EFFECT OF EXERCISE OR MENTAL AND SOCIAL ACTIVITIES ON INTRAINDIVIDUAL VARIABILITY OF COGNITIVE PERFORMANCE IN STROKE

**DOI:** 10.1093/geroni/igae098.3478

**Published:** 2024-12-31

**Authors:** Vrinda Dimri, Nárlon Cássio Boa Sorte Silva, Guilherme Balbim, Janice Eng, Teresa Liu-Ambrose

**Affiliations:** University of British Columbia, Vancouver, British Columbia, Canada; University of British Columbia, Vancouver, British Columbia, Canada; University of British Columbia, Vancouver, British Columbia, Canada; University of British Columbia, Vancouver, British Columbia, Canada; University of British Columbia, University of British Columbia, British Columbia, Canada

## Abstract

Stroke is associated with increased risk of cognitive impairment. Evidence has shown exercise to be a useful strategy for improving cognitive function in this population. Intraindividual variability (IIV), a sensitive measure of cognitive performance, is the within-person trial-to-trial variation in reaction time during cognitive tasks. Whether exercise training also improves IIV of cognitive performance in stroke is unknown. The study was a six-month single-blinded, 3-group parallel randomised controlled trial. Participants included community dwelling adults (N= 119, 38.7% female) with a history of stroke, aged above 55 years (mean= 70.71, SD= 8.59), able to walk 6 meters, and without dementia. Participants were randomly allocated to twice-weekly supervised classes of 1) exercise training (EX); (2) cognitive and social activities (ENRICH); or (3) a balance and tone (BAT; control). Residualised intraindividual standard deviation (rISD) was used as measure of IIV and computed using trial latencies of a computerised Stroop Task. IIV was assessed at baseline, 6-and-12 months. At 6-and-12 months, there was no significant reduction in IIV for all three groups. Furthermore, there were no significant differences in the IIV scores of the EX and BAT (estimated mean difference= 0.30, SE= 0.53, p-value = 0.89) or ENRICH and BAT (estimated mean difference= 0.64, SE= 0.55, p-value = 0.40) groups. The findings suggest that a 6-month program of exercise or mental and social activities does not significantly impact IIV of cognitive performance in stroke. Further studies are required to determine if IIV can be improved using other types of exercise or cognitive training programs.

